# Status of occupational protection in the COVID-19 *Fangcang* Shelter Hospital in Wuhan, China

**DOI:** 10.1080/22221751.2020.1803145

**Published:** 2020-08-12

**Authors:** Min Zhang, Lili Wang, Siyuan Yu, Guixin Sun, Han Lei, Wenjuan Wu

**Affiliations:** a Department of Laboratory Medicine, Shanghai East Hospital, Tongji University School of Medicine, Shanghai, People’s Republic of China; b Department of emergency rescue, Shanghai East Hospital, Tongji University School of Medicine, Shanghai, People’s Republic of China; c Department of Respiration, Shanghai East Hospital affiliated to Tongji University, Shanghai, People’s Republic of China

**Keywords:** COVID-19, SARS-CoV-2, health-care workers, *Fangcang* Shelter Hospital, occupational protection

## Abstract

Staff and employees “Zero infection” has been achieved during the whole medical activities in the COVID-19 *Fangcang* Shelter Hospital in Wuhan, China. This study analyses the personnel and environmental protection status of the East–West Lake *Fangcang* Shelter Hospital. The HCWs were mostly composed of national medical rescue teams, from different provinces in China. Before the COVID-19 outbreak, 82.64% of the HCWs had already known the proper procedure of wearing masks and other personal protective equipment (PPE). For the total of 634 participants entering the inpatient areas, 99.8% of them took occupational protection trainings via various methods. By carefully training and supervision, most of them were competent to work in the inpatient areas six hours/d, three-four times/week. Besides, 7.8% experienced different types of occupational exposure, which mainly caused by the damage of PPE. Once exposed, the HCWs would disinfect skin or mucous in time. No SARS-CoV-2 RNA was detected in 48 air and environmental samples after regular disinfection and cleaning. To conclude, the bundle including intensive training, strengthened personal protection, strict environmental disinfection and timely remedial measures for occupational exposure had ensured the safety of the East–West Lake *Fangcang* Shelter Hospital.

## Introduction

In early February 2020, with the shortage of beds available for the treatment of viral infections in Wuhan, China, there were thousands of patients with mild to moderate COVID-19 sent home for isolation and observation. However, home isolation could put patients’ family members at risk. Therefore, a total of sixteen *Fangcang* shelter hospitals were built rapidly in different districts of Wuhan, to treat and isolate the patients with mild to moderate COVID-19, by converting exhibition centres and stadiums. Although there was some difference between these *Fangcang* shelter hospitals, all of the hospitals were supervised by infection control experts, and qualified health-care workers (HCWs) were employed there.

Compared to general hospitals or hospitals for infectious diseases, staff working in the Fangcang shelter hospitals was complexed. In addition to the activities directly participated by doctors and nurses, there were police officers, housekeeping, sanitation workers, and nucleic acid laboratory workers in the P3 laboratory. Therefore, the corresponding PPE requirements were established according to the biosafety risk level. For example, the nucleic acid and blood test personnel, sputum suction and respiratory tract sampling personnel, and the garbage removal/terminal disinfection personnel were protected in biosafety level 3 – PPE (GB19082-2009, EN14126), N95 masks, isolation gowns, goggles, face shields, long shoe covers or alternatives (e.g. thick plastic bags, rain boots), double medical gloves, waterproof apron/long gloves/long rain boots/splash screen (for garbage and excreta handlers); Doctors, CT testing personnel, non-sampling nurses, work/police maintenance support staff, and severe patient transfer staff were protected in level 2 – PPE (EN 14605 type3/type 4, ISO 13982-1&2 type5), goggles or face shield (difference compared with level 3); Catering/drinking water carrier, specimen transshipment staff, medicine dispensers who did not enter the Fangcang shelter hospitals were protected in enhanced level 1 – isolation or chemical protective clothing, N95 or medical surgical masks, goggles, and gloves; Outside management staff were protected in level 1 – isolation or work clothes, medical surgical masks, gloves and hats (Details of the criteria are shown in Supplementary file eTable 1).

The East–West Lake Fangcang shelter hospital was the first to be built, with 1760 confirmed patients admitted from February 7 to March 8, 2020. A total of 1169 HCWs were employed there, including 144 doctors, 839 nurses and 186 management and security personnel. “Zero infection” of HCWs was achieved. With the stabilization of the epidemic and the decrease of bed occupancy, all of the sixteen Fangcang shelter hospitals were closed by March 10, 2020. With the current lack of PPE globally and pressure on healthcare systems worldwide, the protection of HCWs is becoming an increasing concern. In this article, we focused on the occupational protection and environmental protection status of the East–West Lake *Fangcang* shelter hospital.

## Materials and methods

### Questionnaire

A cross-sectional study design was used. A questionnaire was sent to all 1169 HCWs by the management staff of the East–West Lake *Fangcang* shelter hospital via Web. They kept reminding the HCWs weekly to fill out the questionnaire from February 21 to March 8, 2020. In order to ensure the authenticity of the results, the HCWs were asked to fill out the questionnaire anonymously and voluntarily. The questionnaire was originally designed by the infection control team of the East–West Lake *Fangcang* shelter hospital based on the requirements of the infection control and personnel situation. It consisted of three sections: sociodemographic characteristics, occupational protection characteristics of HCWs at East–West Lake *Fangcang* shelter hospital, and occupational protection characteristics of HCWs entering inpatient areas. The original link of the questionnaire is available: https://www.wjx.cn/mobile/statnew.aspx?activity=58993199&reportid=#1 (Chinses version).

### Environmental air and surface monitoring

**Figure 1.**
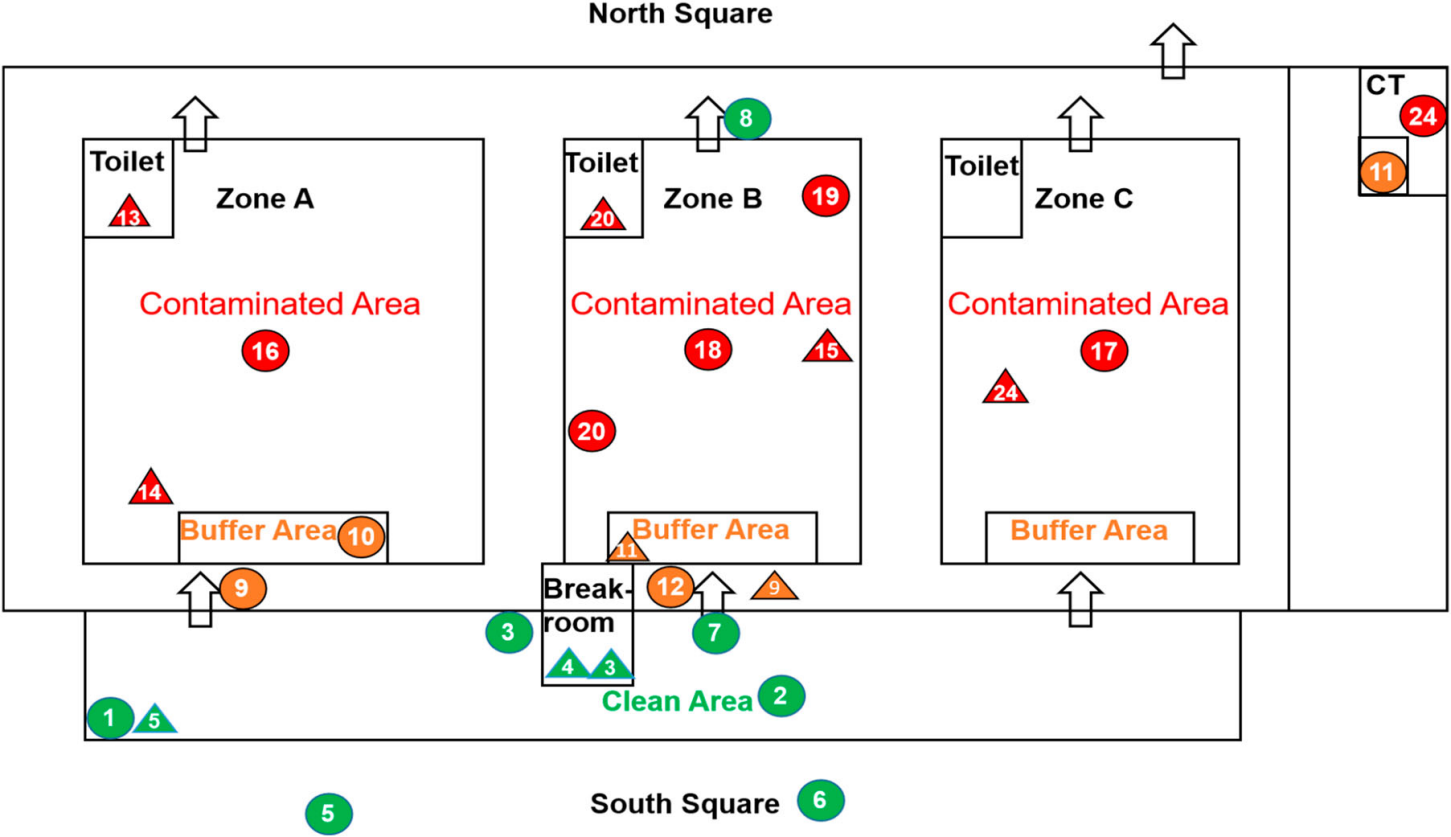
Layout Showing Environmental and Air Sampling Sites. Numbered labels correspond to environmental and air sampling sites listed in Supplementary File eTable 2. Green, yellow and red represent clean area, buffer area and contaminated area, respectively. Circles and triangle represent air and environmental sampling sites, respectively. Arrows show entrance and exit positions. Repeated measurement sites are not shown.

Air sampling was performed on four days using Air Virus collection equipment (NingBo iGene Tec^TM^) with a 0.1 μm gelatin membrane filter for 10 min at 6 m^3^/h. The surface of the environmental object was sampled using a swab, and placed in a virus preservation solution for transportation. Samples were detected daily in a biosafety level 3 (BSL-3) laboratory (Peking Union Medical College Hospital, Chinese Academy of Medical Sciences) by polymerase chain reaction (PCR) testing (BGI Europe A/S kit, China) conducted within 24 h. The BGI kit was on the WHO emergency use listing for in vitro diagnostics detecting SARS-Cov-2 nucleic acid. The target gene to be amplified was ORF1ab. A total of 20 μL reaction buffer was set up containing 1.5 μL enzyme-mix and 18.5 μL reaction buffer in BGI^TM^ SARS-CoV-2 One-step Quantitative RT–PCR system. Thermal cycling was performed at 50°C for 20 min followed by an initial denaturation at 95°C for 10 min and 40 cycles of amplification at 95°C for 15 s and 60°C for 30 s. If the FAM channel amplification curve of the sample to be tested was an S-shaped curve (significant exponential growth period), and the Ct value was ≤38, then the nucleic acid result was supposed to be positive (The layout of the environment and air sampling locations is shown in Figure 1, Supplementary File eTable 2.).

### Evaluation of COVID-19 infection status in HCWs

According to the regulations of the Chinese Health and Safety Committee, all of the 1169 HCWs were quarantined for 14 days after the end of the work, during which time two whole blood and plasma SARS-CoV-2 antibody tests were performed, along with SARS-CoV-2 RNA detection of pharyngeal and nasopharyngeal swabs, and CT imaging of the lungs. A commercial reagent (Wondfo SARS-CoV-2 Antibody Test [Lateral Flow Method] Catalog No.: W195) approved by the CFDA and CE was used for SARS-CoV-2 antibody detection. According to the instruction, the sensitivity and specificity of the antibody kit were 86.43% and 99.57%. The positive predictive value and negative predictive value of it were 99.7% and 82.7%. The SARS-CoV-2 RNA detection method was the same as that described above.

### Data analysis

The Statistical Package for the Social Sciences software (SPSS version 20.0; IBM Corporation, Armonk, NY, USA) was used for data analysis. Sociodemographic data and occupational characteristics data were summarized by the frequencies and percentages of occurrence. The chi-square test was used to compare the frequencies of respondents that did or did not experience occupational exposure associated with categorical variables. The variables included age, working years, education level, previous experience in medical rescue, type of masks used daily, the average duration of wearing one mask, residential hotels had proper infection control measures, satisfied with meals, major concerns regarding the current situation, felt discomfort during shifts, felt the polluted air or the unideal temperature, occupational exposure while working in inpatient areas, type of shoes wore in inpatient areas, extent the protective gears impacted on efficiency, and strictly followed the infection control procedure about putting on/taking off protection clothing every time entering/leaving inpatient areas. The validity of these variables was 0.698. For all statistical analyses, a *P* value of ≤0.05 was considered significant.

## Results

### Sociodemographic characteristics

A total of 1169 HCWs worked at the East–West Lake *Fangcang* shelter hospital, including 144 doctors, 839 nurses and 186 management and security personnel. There were 645 participants of them completed the questionnaire anonymously and voluntarily. Among the 645 HCWs, the majority (93.90%) were from provinces other than Hubei province. They comprised 140 (21.71%) males and 505 (78.29%) females, with ages ranging from 22 to 56 years (average 33.98 ± 6.701 years), and the number of working years ranging between 0.5 and 39 (average 11.82 ± 6.9511years). More than half (64.8%) were from tertiary hospitals, and almost all had achieved high-level education (66.67% undergraduate, 5.12% master’s degree or above). Nursing was the most common occupation (80.16%) (Table 1).

**Table 1.** Sociodemographic characteristics of HCWs at the East-West *Fangcang* shelter hospital.

		No. (%)
Gender	Male	140 (21.71)
	Female	505 (78.29)
Age^a^	≤30	224 (34.78)
	>30	420 (65.22)
Working years	≤15	447 (73.95)
	>15	168 (26.05)
Education level	Undergraduate	430 (66.67)
	Master's degree and above	33 (5.12)
	Technical college	164 (25.43)
	Technical secondary school	18 (2.79)
Region^b^	Wuhan, Hubei	39 (6.09)
	Provinces bordering Hubei	119 (18.59)
	Other provinces	482 (75.31)
Rank of the residential hospital	Tertiary	418 (64.81)
	Secondary	225 (34.88)
	Primary	2 (0.31)
Job title	Physician	48 (7.44)
	Nursing	517 (80.16)
	Technician	14 (2.17)
	Administrative	41 (6.36)
	Security guard	2 (0.31)
	Others	23 (3.57)
Working department	Internal medicine	244 (37.84)
	Respiratory	69 (10.70)
	Cardiology	31 (4.81)
	Infectious Diseases	25 (3.88)
	Surgery medicine	100 (15.51)
	Department of Critical Medicine	80 (12.40)
	Emergency Department	53 (8.22)
	Pediatrics	20 (3.10)
	Gynecology	22 (3.41)
	Other clinical departments	73 (11.32)
	Clinical assistant department	15 (2.33)
	Administration	3 (0.47)
	Infection control department	4 (0.62)
	Operation support department	31 (4.81)
Ethnicity	Han	611 (94.73)
	Hui	25 (3.88)
	Others	9 (1.40)
Number of days had been working in Hubei so far^a^	≤3 weeks	536 (83.23)
	>3 weeks	108 (16.77)

^a^Missing one sample.

^b^Missing five samples.

### Occupational protection characteristics

**Figure 2.**
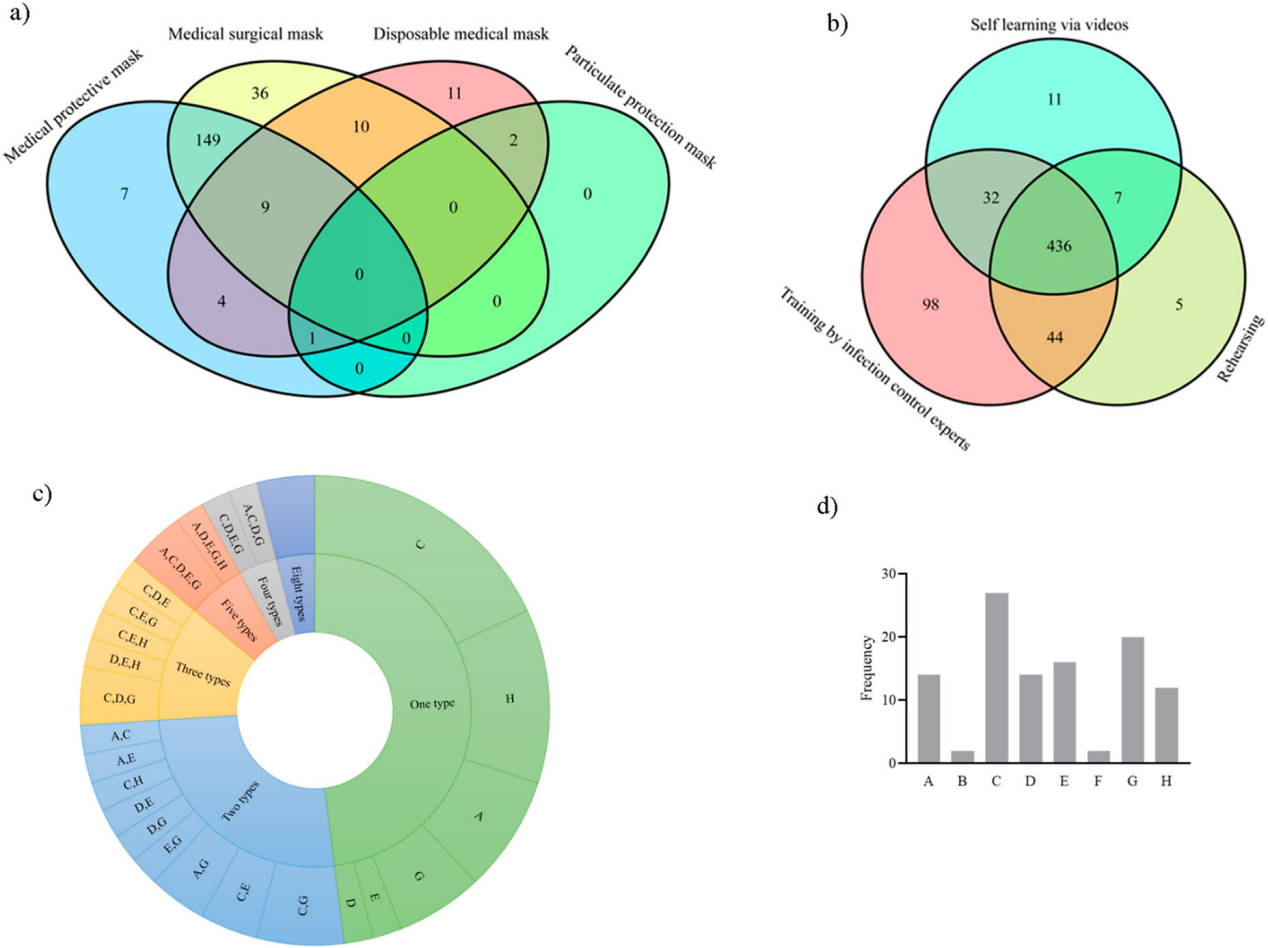
Occupational protection characteristics of HCWs according to the questionnaire. (a) Types of multiple masks worn by the HCWs (*n* = 229), (b) types of infection control training methods undertaken by the HCWs before entering the inpatient areas (*n* = 633), (c) types of occupational exposure experienced by the HCWs (*n* = 50), (d)the frequency with which each kind of occupational exposure occurred among the 50 HCWs (A-Dropped masks, B-Exposed to secretions without facial protection, C-Damaged protective suits, D-Damaged gloves, E-Damaged shoe covers and shoes, F-Needle stick injury, G-Exposure when taking off PPE, H-Others).

Before the COVID-19 outbreak, 18.29% of the 645 HCWs had previous experience in medical rescue, and only 0.9% were inexperienced in the proper procedure for wearing masks. Medical surgical masks were the most common type of masks (61.09%) used by the HCWs during their daily work in the East–West Lake *Fangcang* shelter hospital, followed by disposable medical masks (27.60%) and medical protective masks (10.23%). Notably, particulate protection masks were also used (1.09%) (Table 2). Only approximately one-third of the HCWs (35.50%) wore multiple masks. Among them, 65.07% used medical protective masks together with medical surgical masks, and 15.72% used a double layer of medical surgical masks, while 1.31% used particulate protection masks together with disposable medical masks or medical protective masks (Figure 2). About half of the HCWs (54.26%) changed their masks every 4 h, 36.43% every 6–8 h and 9.31% of HCWs changed their masks every 12 h or even longer. Most HCWs (90.69%) entered inpatient areas no more than four times a week, while others entered 5–6 times (8.53%) or more than six times (0.78%).

**Table 2:** Occupational protection characteristics of HCWs at the East-West Fangcang shelter hospital (*n* = 645).

		No. (%)
Previous experience in medical rescue	Yes	118 (18.29)
	No	527 (81.71)
Occupation during previous medical rescue (*n* = 118)	Medical staff	87 (73.73)
	Medical technician	5 (4.24)
	Administrator	13 (11.02)
	Guard	1 (0.85)
	Others	12 (10.17)
Knew about proper procedure of wearing masks	Yes	533 (82.64)
	Know some	106 (16.43)
	No	6 (0.93)
Type of masks used daily^a^	Medical surgical mask	394 (61.09)
	Disposable medical mask	178 (27.60)
	Medical protective mask	66 (10.23)
	Particulate protection masks	7 (1.09)
Wore multiple masks	Yes	229 (35.50)
	No	416 (64.50)
Average duration of wearing one mask^b^	4 h	350 (54.26)
	6–8 h	235 (36.43)
	12 h	17 (2.64)
	24 h	21 (3.26)
	>24 h	22 (3.41)
Frequency of entering the inpatient area (times/week)	0	72 (11.16)
	1–2	312 (48.37)
	3–4	201 (31.16)
	5–6	55 (8.53)
	≥7	5 (0.78)
Living environment	Single room	307 (47.60)
	Twin room	337 (52.25)
	Multi-person living room	1 (0.16)
Residential hotels had proper infection control measures	Yes	608 (94.26)
	No	37 (5.74)
Satisfied with meals	Yes	572 (88.68)
	No	6 (0.93)
	Sometimes no	67 (10.39)
Major concerns regarding the current situation	Personal protection against the virus	550 (85.27)
	Safety of the residential environment	34 (5.27)
	Mental health	22 (3.41)
	Leisure	31 (4.81)
	Dining	8 (1.24)
The most common infection source considered by the HCWs	In the Fangcang shelter hospital	276 (68.66)
	Exposure while taking off the PPE	85 (21.14)
	Lack or damage of PPE	66 (16.42)
	Contact patients closely	20 (4.98)
	Others	105 (26.12)
	Out of the Fangcang shelter hospital	71 (17.66)
	Twin room	40 (9.95)
	Crowded place	18 (4.48)
	Others	13 (2.23)
	Personal factors	55 (13.68)
	Inappropriate self-protection	36 (8.96)
	Fatigue	11 (2.74)
	Decreased immunity	7 (1.74)
	Mental health	1 (0.25)

^a^The filtration efficiency for 0.3 micron particles of medical protective masks (N95/KN95 masks for medical usage)and particulate protection masks (N95/KN95 masks for industry usage) were ≥95%, The filtration efficiency for 0.3 micron particles and 3 micron particles (e.g. bacteria) of medical surgical masks was ≥30% and ≥95%. The filtration efficiency of disposable medical masks were unknown, but lower than the medical surgical masks.

^b^The recommended time for each mask was 4 h.

Of the 634 participants entering inpatient areas, only one, who worked as a driver, had not undergone any occupational protection training. When the driver delivered the supply of materials to the East–West Lake *Fangcang* shelter hospital for the first time, he handed over the materials to the nurse in the semi-contaminated area, which actually means that he did not enter the inpatient areas. After the situation was found, he was trained in time. Apart from that, the rest had undergone training via various methods (Figure 2, Table 3). Similarly, one of the HCWs, who worked as a nurse, did not follow the infection control procedure to put on/take off PPE on entering/leaving inpatient areas. Despite undergoing training through all three methods, this individual was confused by the lack of professional guidance from a dressing room instructor (data not shown). A minority of the HCWs (8.36%) wore their own shoes in inpatient areas. Notably, 50 of the HCWs (7.89%) experienced various types of occupational exposure while working in inpatient areas. Among them, more than one half experienced more than one type of occupational exposure. The percentage of HCWs who felt discomfort during shifts, were anxious about breathing polluted air or found the temperature difficult to tolerate, and who agreed that PPE had a large impact on their work, was higher among those that had undergone occupational exposure compared with those who had not (Table 4). The types of occupational exposure, ranked by cumulative frequency, were damaged protective suits (27), exposures when taking off PPE (20), damaged shoe covers and shoes (16), dropped masks (14) and damaged gloves (14).

**Table 3.** Occupational protection characteristics of HCWs in inpatient areas (*n* = 634).

		No. (%)
Training experience before entering	Yes	633 (99.84)
	No	1 (0.16)
Felt discomfort during shifts	Often	78 (12.30)
	occasionally	412 (64.98)
	No	144 (22.71)
Felt the polluted air or the unideal temperature	Yes	241 (38.01)
	No	393 (61.99)
Occupational exposure while working in inpatient areas	Yes	50 (7.89)
	No	584 (92.11)
Type of shoes wore in inpatient areas	Nurse shoes	294 (46.37)
	Rubber shoes	287 (45.27)
	Own shoes	53 (8.36)
Extent the protective gears impacted on efficiency	Largely	199 (31.39)
	Some	325 (51.26)
	Basically able to adapt	110 (17.35)
Strictly followed the infection control procedure about putting on/taking off protection clothing every time entering/leaving inpatient areas	Yes	633 (99.84)
	No	1 (0.16)
Major concerns regarding the current situation	Quantity, quality, comfort of PPE	133 (51.55)
	Trainings on occupational exposure emergency treatment	57 (22.09)
	Facilities	21 (8.14)
	Cleaning, ventilation and air monitoring	16 (6.20)
	Hand hygiene	8 (3.10)
	Physical and mental health	10 (3.88)
	Others	13 (5.04)

**Table 4.** Occupational characteristics compared between non-occupational exposure and occupational exposure of HCWs.

		No. (%)		*P* value
		non-exposure (*n* = 584)	exposure (*n* = 50)	
Felt discomfort during shifts	Often	66(11.30)	12(24.00)	0.002
	Occasionally	377(64.55)	35(70.00)	
	No	141(24.14)	3(6.00)	
Felt the polluted air or the unideal temperature	Yes	210(35.96)	31(62.00)	<0.001
	No	374(64.04)	19(38.00)	
Major concerns regarding the current situation	Personal protection against the virus	501(85.79)	40(80.00)	0.008
	Safety of the residential environment	33(5.65)	0(0)	
	Mental health	18(3.08)	4(8.00)	
	Leisure	27(4.62)	3(6.00)	
	Dining	5(0.86)	3(6.00)	
Residential hotels had proper infection control measures	Yes	555(95.03)	43(86.00)	0.002
	No	29(4.97)	7(14.00)	
Extent the protective gears impacted on efficiency	Largely	171(29.28)	28(56.00)	<0.001
	Some	305(52.23)	20(40.00)	
	Basically able to adapt	108(18.49)	2(4.00)	

^a^Occupational exposure was supposed to occur when the following conditions happened: dropped masks, damaged protective suits, damaged shoe covers and shoes, damaged gloves, exposure when taking off PPE, needle stick injury, exposed to secretions without facial protection, etc.

Among the 645 participants, the major concern regarding their work was personal protection (85.3%). Additionally, the 50 HCWs who had experienced occupational exposure were concerned about mental health, dining and leisure (Table 4). The most common infection sources among the HCWs were supposed to include exposure while taking off PPE (21.14%), lack or damage of PPE (16.42%), close contact with patients (4.98%), as well as personal factors like inappropriate self-protection (8.96%), fatigue (2.74%), and decreased immunity (1.74%). Areas needing improvement included the quantity, quality, and comfort of PPE (51.55%); training on occupational exposure during emergency treatment (22.09%); facilities (8.14%); and cleaning, ventilation and air monitoring (6.20%). Furthermore, nearly half of the HCWs (52.41%) lived in twin or multi-person living rooms, and most of the residential hotels (94.26%) had proper infection control measures.

### Environmental air and surface monitoring

A total of 48 air and environmental surface samples were collected over four days from February 26 to March 7. All PCR results were negative for SARS-CoV-2 RNA. That is, the 2019 SARS-COV2 nucleic acid values in the samples are lower than the detection limit of the BGI^TM^ SARS-CoV-2 RT–PCR system (100copies/mL).

### Evaluation of the COVID-19 infection status among HCWs

All HCWs were quarantined for 14 days after finishing their work, during which time, two whole blood and plasma SARS-CoV-2 antibody tests, SARS-CoV-2 RNA detection of pharyngeal and nasopharyngeal swabs, and CT imaging of the lungs were performed. All of the results were negative.

## Discussion

At present, the global COVID-19 epidemic is ongoing [1]. Traditional hospitals in many countries are unable to provide sufficient beds for the increasing number of infected patients. In the early stage of the COVID-19 epidemic in China, *Fangcang* shelter hospitals provided purpose-built places for the isolation and treatment of patients with mild and moderate symptoms, effectively promoting the control of the Chinese epidemic [2,3].

Although there were a large number of confirmed patients in the East–West Lake *Fangcang* shelter hospital, efficient infection control practices were undertaken by HCWs. Firstly, before entering the East–West Lake *Fangcang* shelter hospital, the HCWs received the latest information about symptom onset, transmission and standard procedure of daily practices, through various types of educational methods [7]. Similarly, other studies have reported the significant role of training on reducing the risk of infection [4–6,8]. We recommend that training and supervision for drivers, security personnel, cleaners, as well as instructors, need to be strengthened. Secondly, HCWs selected appropriate PPE, and applied and removed this PPE, following the standard guidelines. As previously stated, the improvements in PPE are required to increase comfort for HCWs [8–10], and experience in selecting the correct size of PPE, decreased the risk of damage [7]. Thirdly, effective emergency measures were developed. If PPE was damaged, and if there was no direct contact with the inner clothing and skin, the HCWs were asked to wash hand, change and replace with a new PPE immediately, as well as doff and exit ward according to the protocol after work. If the skin was exposed or damaged by sharp things, the HCWs were asked to clean the skin immediately with iodine and 75% alcohol. If mucous membranes (mostly the eyes) were exposed, the HCWs should rinse eyes with saline immediately. One shall report to the head nurse, and immediately doff and exit ward according to the protocol, as well as report the incident to leaders in charge, and arrange self-quarantine or isolation for observation. These measures ensured the safety of the HCWs who had experienced occupational exposure. Notably, our data raised concerns regarding the physical and mental health of exposed HCWs [11]. Finally, appropriate infection control measures were also conducted in the HCWs’ living accommodation, with the physical division being implemented in double rooms.

Additionally, strict cleaning procedures were applied to environmental surfaces and air disinfection measures were performed. Environmental contamination and airborne droplets have been proposed as common routes of transmission [12]. In the hospital, surfaces, such as floors and those in contact with patient belongings, were soaked, sprayed or scrubbed with 1000 mg/L chlorine-containing disinfectant, with an effect lasting > 30 min, at least 4 times/d. Similar to previous reports, after regular disinfection and cleaning, environmental samples tested negative following nucleic acid detection [1,13]. In addition, patients in the East–West Lake *Fangcang* shelter hospital were asked to wear masks at all times, and change their masks once daily. They were also educated to stop spitting around. Air purification and disinfection apparatus were operated. These measures help to decrease the risk of infection via airborne droplets [12]. It is worth nothing that although one study reported a positive result from an environmental sample from a patient toilet at a *Fangcang* shelter hospital, only negative results were obtained after rigorous sanitization [14].

This study has some limitations. Firstly, due to the fact that the public traffic in Wuhan was interrupted and communication was blocked at the time of our investigation, it was too challenge to conduct a multi-centre study with other *Fangcang* shelter hospitals. Secondly, in the design of the questionnaire survey, some characteristics such as suggestions for the infection control measures and feelings after occupational exposure can be more detailed. Thirdly, the guiding role of research results in subsequent infection control measures should be strengthened.

To conclude, the protection of HCWs during the COVID-19 epidemic is crucially important. There were approximately 3000 HCWs got infected in the early epidemic in China. Although the East–West Lake *Fangcang* shelter hospital admitted 1760 confirmed patients, none of the 1169 HCWs has infection symptoms so far. Overall, a variety of measures including intensive training, strengthened personal protection, thorough environmental disinfection and timely remedial measures following occupational exposure, all contributed to ensuring the safety of HCWs at the East–West Lake *Fangcang* shelter hospital.

## Supplementary Material

Supplementary_Online_Content_clean.docx
