# Supplementary material for: Status of occupational protection in the COVID-19 Fangcang Shelter Hospital in Wuhan, China
Source: Emerg Microbes Infect. 2020 Aug 12;9(1):1835–42. doi: 10.1080/22221751.2020.1803145 (PMC7473240; doi:10.1080/22221751.2020.1803145)
Supplement: Supplementary_Online_Content_clean.docx [file TEMI_A_1803145_SM3071.docx]

**Supplementary Online Content**

**eTable 1. Guidance on PPE for the *Fangcang* shelter hospital staff**

**eTable 2. Layout Showing Environmental and Air Sampling Sites**

**eTable 1. Guidance on PPE for the *Fangcang* shelter hospital staff**

| **Duty** | **Protection grade** | **PPE** | **Mask** | **Other** |
| --- | --- | --- | --- | --- |
| Nucleic acid and blood test personnel | Level 3 | GB19082-2009; EN14126 | GB19083-2010 (N95) | Isolation gowns; Goggles and face shields; Long shoe covers or alternatives (e.g. thick plastic bags, rain boots); Double medical gloves; Garbage and excreta handlers need waterproof apron/long gloves/long rain boots/splash screen |
| Sputum suction and respiratory tract sampling |  |  |  |  |
| Garbage removal/terminal disinfection personnel and people who have a lot of contact with dirt such as feces, vomit, etc |  |  |  |  |
| Doctor/CT | Level 2 | EN 14605 type 3/type 4; ISO 13982-1&2 type 5 | GB19083-2010 (N95) | Isolation gowns; Goggles or face shields; Long shoe covers or alternatives (e.g. thick plastic bags, rain boots); Double medical gloves |
| Non-sampling nurse |  |  |  |  |
| Work/Police/maintenace support staff |  |  |  |  |
| Severe patient transfer staff |  |  |  |  |
| Catering/drinking water carrier (do not enter the hospital) | Level 1 (enhanced)^a^ | Isolation or chemical protective clothing | GB19083-2010 (N95) or medical surgical mask | Goggles; Gloves |
| Specimen transshipment staff (do not enter the hospital) |  |  |  |  |
| Medicine dispensers (do not enter the hospital) |  |  |  |  |
| Outside management staff | Level 1 | Isolation or work clothes | Medical surgical mask | Gloves; Hat |

^a^Although the catering/drinking water carrier, external specimen transfer personnel and medicine delivery personnel do not enter the cabin, they have handed over to the personnel receiving the goods in the cabin. In order to avoid accidents during operation, primary protection is set to protect the eyes and respiratory tract.

**eTable 2.** **Layout showing environmental and air sampling sites**

| **Area** | **Air Sampling** | | | **Environmental Sampling** | | |
| --- | --- | --- | --- | --- | --- | --- |
|  | **No.** | **Sampling Time** | **Sampling Sites** | **No.** | **Sampling Time** | **Sampling Sites** |
| Clean Area | 1 | 2020/2/26 | Plaza Command Center | 1 | 2020/3/7 | Zone B Entrance  Workbench |
|  | 2 | 2020/2/27 | Clean Area Square | 2 | 2020/3/7 | Zone B  Dustbin, External Rim |
|  | 3 | 2020/2/27 | Corridor of Zone A and B | 3 | 2020/3/7 | Zone B Female Breakroom  Cabinet Handle and Key |
|  | 4 | 2020/3/7 | Corridor of Zone A and B | 4 | 2020/3/7 | Zone B Male Breakroom  Back of the chair |
|  | 5 | 2020/3/7 | Square Entrance | 5 | 2020/3/7 | Command Center (Clean Area)  Computer mouse |
|  | 6 | 2020/3/7 | Command Center Square | 6 | 2020/3/7 | Command Center (Clean Area)  Keyboard |
|  | 7 | 2020/3/7 | Zone B Entrance | 7 | 2020/3/7 | Command Center (Clean Area)  Tap |
|  | 8 | 2020/3/7 | Zone B Export | 8 | 2020/3/7 | Command Center (Clean Area)  Desktop |
| Buffer Area | 9 | 2020/2/26 | Zone A Protective Apparel Removal  Room (PARR) | 9 | 2020/3/6 | Zone B Protective Apparel Removal Room 1 (PARR 1)  Desktop |
|  | 10 | 2020/2/26 | Zone A Buffering Room | 10 | 2020/3/6 | Zone B Protective Apparel Removal Room 2 (PARR 2)  Desktop |
|  | 11 | 2020/2/27 | CT Buffering Room | 11 | 2020/3/6 | Zone B Buffering Room  Desktop |
|  | 12 | 2020/3/6 | Zone B Protective Apparel Removal Room 1 (PARR 1) |  |  |  |
|  | 13 | 2020/3/6 | Zone B Protective Apparel Removal Room 2 (PARR 2) |  |  |  |
|  | 14 | 2020/3/6 | Zone B Buffering Room Entrance |  |  |  |
| Contam-inated Area | 15 | 2020/2/26 | Samples Collection Room | 12 | 2020/3/6 | Zone A Nurse Station  Keyboard |
|  | 16 | 2020/2/26 | Zone A Patient’s Room | 13 | 2020/3/6 | Zone A Toilet  Door |
|  | 17 | 2020/2/26 | Zone C Patient’s Room | 14 | 2020/3/6 | Zone A  TV screen |
|  | 18 | 2020/2/26 | Zone B Patient’s Room | 15 | 2020/3/6 | Zone B Patient’s Activity Area  Desktop |
|  | 19 | 2020/3/6 | Zone B Patient Bedside | 16 | 2020/3/6 | Zone B Samples Collection Room  Desktop |
|  | 20 | 2020/3/6 | Zone B Patient Activity Area | 17 | 2020/3/6 | Zone B  Patient's Bench |
|  | 21 | 2020/3/6 | Zone B Patient Mobile Toilets Area | 18 | 2020/3/6 | Zone B Surface of Patient’s Bedside Table |
|  | 22 | 2020/3/6 | Zone B Doctor’s Workstation | 19 | 2020/3/6 | Zone B Surface of Patient’s Mask |
|  | 23 | 2020/3/6 | Zone B Samples Collection Room | 20 | 2020/3/6 | Zone B Toilet  Door Handle |
|  | 24 | 2020/3/7 | CT Examination Room | 21 | 2020/3/6 | Zone B  Patient’s Bed Rail |
|  |  |  |  | 22 | 2020/3/6 | Zone B Doctor Workstation  Computer Screen |
|  |  |  |  | 23 | 2020/3/6 | Zone B Doctor Workstation  Keyboard |
|  |  |  |  | 24 | 2020/3/6 | Zone C Surface of Patient’s Bedside Table |
